# An Inquiry, Critical and Experimental, into the Pathology of Fever

**Published:** 1853-06

**Authors:** N. S. Davis

**Affiliations:** Professor of Pathology, Practice of Medicine, and Clinical Medicine, in Rush Medical College; and one of the Physicians of the Ill. Gen. Hospital


*An Inquiry, Critical and Experimental, into the Pathology of Fever.* By N. S. Davis M. D. Professor of Pathology, Practice of Medicine, and Clinical Medicine, in Rush Medical College; and one of the Physicians of the Ill. Gen. Hospital.

[Continued from Page 32.]

In thirty minutes more the temperature was found the same, the pulse 72, and respiration 16, with a further diminution of natural feelings. The exhilaration in the head, had now become converted into a sensation of tightness and dull pain. At each time of noting the temperature, pulse &c., a given quantity of *expired* air was collected and the quantity of carbonic acid in it ascertained. During the first hour after taking the brandy, the relative proportion of carbonic acid exhaled, underwent a rapid and marked diminution. At the end of two and a half hours, it was fully equal to the proportion before the experiment began. In all the examinations of temperature, the bulb of the thermometer was inserted under the tongue, with the mouth closed around it for ten minutes, and the height of the mercury noted by a careful assistant before its removal.

*Experiment* 2.—Nov. 8., 1852, 9 o'clock P. M., in the same room, with the temperature, regulated in the same *manner* as in experiment number 1. Three hours after a light supper, my temperature under the tongue was $97\frac{3}{4}$ F., pulse 72, and respirations 18 per minute. I drank at once 8 *ounces of good* old Port Wine. In *thirty* minutes after drinking the wine, my temperature was $97\frac{5}{8}$ F., pulse 78, respirations 19.

Ten o'clock P. M., one hour after taking the wine, feel much exhilaration of mind and brain, eyes hot, mouth and fauces dry, a sense of numbness in the hands, face and throat; muscular weakness or a feeling as though I should stagger without an effort to prevent it. Temperature $97\frac{3}{8}$° F., pulse 77, and respiration 19.

Eleven o'clock, two hours after the wine, same unnatural feelings continue, with more approach towards head-ache and a sense of dullness and tightness above the ears. Temperature $97\frac{1}{8}$° F., pulse, 73, and respiration 19.

Twelve o'clock, three hours after the wine, mouth less dry, but a dry bad feeling in the throat, less exhilaration and more pain, with

Reel 178-10-5 CONT.

heaviness in the head, and some general feeling of lassitude.—Temperature 97° F., pulse 68, and respiration 17.

The effect on the relative proportion of carbonic acid in the expired air was the same in this as in the preceding experiment. In both experiments other qualities of the pulse besides mere frequency, were carefully noted, and the gentleman who assisted me, thought that its fullness and firmness was diminished in pretty close proportion to its increase in frequency. The excretory evacuations were also noted carefully during the 24 hours following each experiment, but no change was observable, except a slight diminution in the quantity, with a deeper red color, of the urine. Its specific gravity and chemical composition were not examined.

These experiments present us with two distinct and very interesting classes of phenomena. The first embraces the sense of exhilaration in the brain, the general feeling of heat and numbness, and the increased frequency of the pulse; and arise evidently from the direct irritating effects of the alcohol on the nerve structures and the inner surface of the heart and arteries. The second class includes the marked diminution of animal heat; the diminished secretion from the mouth, fauces, and all other secreting structures; and the muscular weakness or diminished power of endurance.

These phenomena, existing conjointly, can only arise from such a modification of the inherent vital properties of the tissues, as to retard their metamorphosis from which carbonic acid is furnished for expiration, caloric evolved for maintaining animal temperature, and the capillary and interstitial circulation maintained in a state favorable for secretion. And the fact that all these elementary organic actions or functions are *depressed*, at the same moment that the brain and nervous system are unequivocally *excited*, affords proof amounting to scarcely less than absolute demonstration, that these functions depend essentially on inherent vital properties, capable of existence and modification. wholly independent of the brain or nerves. If it were otherwise, every excitement or exhilaration of the nervous functions, would necessarily be accompanied by corresponding excitement or increase of calorification, secretion, &c. Equally do the foregoing results demonstrate the fact, that alcoholic drinks taken into the human system, neither support respiration nor animal heat, as represented by Liebig, Carpenter and other chemico-physiologists. On the contrary, they show very clearly that the presence of alcohol in the blood directly excites the nervous tissues and the inner surface of the heart and arteries, inducing mental and nervous exhilaration, temporary excitement of the pulse, and a general glow of heat; while at the same time it so acts on the organic susceptibility and tonicity, or rather molecular affinity, as to suspend or retard those molecular changes by which the tissues undergo metamorphosis, the secretory cells effect their functions, and animal heat is maintained. Hence, when the quantity of alcohol introduced into the human system is too great, its depressing influence on the organic susceptibility so far predominates, that even the nervous tissue fails longer to respond to the presence of the excitement, and the individual becomes wholly unconscious or "*dead drunk*," and sometimes even dies. Whether it produces this direct influence on the the vital properties and organic functions by its strong affinity for the albuminous matter of the tissues, or in some other way, is not material to our present object. The two-fold effects of alcoholic drinks, as above explained, affords a beautiful physiological explanation of their common and almost irresistible influence over the minds and bodies of men.—The first is fascinated and controlled by their exhilarating influence on the brain and nerves; while the second is slowly but certainly impaired in all its functions, by their direct interference with the vital properties and organic actions. Thus, if the quantity taken habitually be small, and especially if it consist of the more dilute beverages, such as wine, porter, beer, &c., it will generally serve simply to retard the metamorphosis or disintegration of the tissues, without materially impairing the processes of assimilation and nutrition, and thereby lead to an accumulation of fat or the adipose tissue, with diminished tonicity and power of endurance. This gives to the individual that *embonpoint*, or fulness of person, redness of face, and indisposition to active physical exertion, which is too frequently seen in all the stages of its development in every part of Christendom. On the other hand, if the quantity taken is large, consisting chiefly of distilled spirits, it sooner or later induces a more profound alteration of the elementary functions. It not only retards disintegration and lessens the proper depuration of the blood by means of the excretory organs, but it also retards and deranges assimilation and nutrition, thereby inducing the same redness and bloating of face, the same impaired condition of vital properties and physical power of endurance, but without any of the *embonpoint*; and sometimes with positive emaciation. The same properties that gives to alcohol the power to exert such influences over the healthy condition of the system, render it a valuable medical agent in certain pathological conditions, characterized by a rapid or excessive disintegration of tisssues and a torpid state of the nervous system. Such a condition is occasionally seen in the advanced stages of tubercular phthisis, and in some of the cases of continued fever.

But in *no case*, do the alcoholic drinks act on the healthy condition of the human system as true *tonics*, by promoting the physical strength, energy, and power of endurance.

The effects of morbific agents, furnish many illustrations of the power to act on the primary properties of the tissues, ihdependent of the nervous system. Thus, while the efficient cause of epidemic cholera leaves the brain and nerves so little disturbed, that all the functions of the mind, with ordinary sensation and motion, continue scarcely impaired; so profound is the depression of organic susceptibility throughout the entire body, that in one brief hour after its work has fairly begun, all nutrition and glandular secretions have ceased; neither the secreting cells nor capillaries respond to the presence of blood in them; the irritability and contractility of the heart itself rapidly diminishes, and the production of animal heat is entirely suspended. That these effects are not the results of excessive evacuations, is proved by the fact that some of the cases in which they occur most rapidly and fatally, are characterized by very little evacuations of any kind.

Regarding these illustrations as sufficient to show that the organic properties are capable of being acted upon by external agents, and that, too, in such a manner as to produce *general* functional disturbance throughout the whole system, it remains to answer the second leading inquiry proposed, viz., whether all the essential phenomena of the different types of fever can be satisfactorily explained, by a knowledge of the effects of different morbific causes on the general vital properties of organized tissues? In answering this question, a few elementary physiological propositions need to be clearly apprehended, and constantly kept in mind.

First, every *vital* act or phenomenon requires for its exhibition three coincident conditions, namely, a definite texture or organization, a susceptability to the action of agents, and the presence of an agent capable of making an impression.

Second, for the exhibition of vital actions of a strictly healthy character, the three foregoing conditions must not only be present, but they must bear a just and proper relation to each other.

For instance, the germinal cells of the fecundated egg may possess the required organization or texture, and the proper susceptibility, but if the stimulus of caloric be not applied in the proper quantity, no healthy development or vital action takes place. On the other hand, if the texture of the germinal cells be changed, or their susceptibility destroyed, no amount of the stimulus of caloric will answer the required purpose. What is true of the simple primary cell, is equally true of the most complex tissues and organs of the human body. So long as every tissue and organ maintains its natural texture, its proper degree of susceptibility, and the presence of its natural stimulus in due proportion, so long will every function be performed with regularity, and health will be preserved. But let anything produce a variation in either of these conditions, and a variation in the functions is a necessary consequence. If anything destroys either, death follows. If the interfering cause is such that it acts only on the conditions or properties of a single tissue or organ, the resulting functional disorder is limited and local; if it acts on the whole at once, the functional disorder is general. But, as we shall soon see, many diseases which are primarily local, from the limited action of the cause, subsequently induce general disturbance from the continued interruption of the function of the part affected.

Third, the human body, regarded as a living physical organization, is composed of numerous tissues and organs, each possessed of a definite texture, and the common elementary property of susceptibility, and holding as its natural stimulant the healthy arterial blood.

While these all remain normal, each organ performs its own specific function with regularity. One group, or class, are employed in elaborating new material to supply the constant waste, which are called digestive and assimilative organs; another, in gathering up the waste, useless, and noxious particles and conveying them out of the system, called excretory organs; another is designed to form a bond of union between the whole, so that any impression of a deleterious nature, made on one part, may call into sympathetic relation all the others. This embraces the nervous system of organic life; while the rest of the nervous matter is connected with the functions of relation.

Fourth, if it is true that the healthy performance of the functions of all the tissues depends on the existence and proper relations of the *three* conditions named in the first proposition, then it legitimately follows that no *general* disease or functional derangement can take place, except through an alteration of one or more of these general conditions.

Such alterations may take place, first, by the direct action of morbid causes or agents on the tonicity and susceptibility of all the tissues; and, second, by changes effected in that universal organic stimulant, the blood; and through it, secondarily also, in the elementary properties of all the tissues in which it circulates. The first class of agents would include the mental emotions, and those imponderable agents, termed caloric, light and electricity; while the second would include all those material agents of a deleterious character, capable of entering the circulating fluid from without, or through the absorbent capillaries, from the metamorphosis of the tissues within, which were not capable of being speedily excreted from the system, on the one hand, or of having their action limited by some special affinity, to a single organ or a group of organs, on the other. It is thus readily perceived that the mutual and proper relation between the tonicity and susceptibility of the solids and the circulating fluid, may be universally disturbed by numerous and diverse agencies, giving rise to equally diverse morbid phenomena and results. For instance, the stronger mental emotions, such as fear, grief, and anxiety, directly impair the *tonicity* of all the tissues, as evidenced by the altered capillary circulation and secretions, the muscular relaxation, the impaired nutrition, &c. These effects, if continued by the persistent action of the cause, sooner or later, induce such changes in the blood and the organic susceptibility as to destroy their natural relations to each other, when that general functional disturbance becomes developed, which constitutes a variety of fever. Again, it has already been stated that a continued exposure to a high temperature diminishes tonicity and increases susceptibility, which, though not generally sufficient of itself to produce the phenomena of active disease, is yet sufficient to constitute that predisposition which is well known to exist during the hot months of the year, and to give rise, on the intervention of exciting causes, to those rapid local determinations which constitute cholera morbus, diarrhœa, dysentery, &c.; or those more general diseases of high irritative excitement, such as the various forms of periodic fevers in malarious districts, and the more active forms of continued fever in other localities. On the other hand, the effects of exposure to a low temperature, in increasing tonicity and diminishing susceptibility, is equally apparent to every observer. At first the condensation of tissues or increased tone that accompanies exposure to a low temperature, imparts a feeling of increased strength and energy; but if the exposure is continued, this soon gives place to stiffness or indisposition to active exertion, and a blunted or obtuse state of all the susceptibilities of the system, accompanied by diminished force in both respiration and circulation. While the organic susceptibility is thus blunted by the direct influence of cold, the molecular changes, by which new matter is deposited in the tissues, and effete matter is returned into the circulation to be removed through the organs of excretion, take place with less freedom and activity, thereby allowing more or less accumulation, in all the tissues, of particles which in the healthy state of the organic action would have been removed.

When the exposure to cold ceases, and a more elevated temperature again restores the healthy state of the vital properties, the presence of these particles of retained matter embarrasses and disturbs the molecular changes, inducing a degree of susceptibility and organic action, as much in excess as there had been previous deficiency. It is thus that sudden and extreme changes of temperature develop local inflammations; while more protracted exposure to moderate cold, from deficient fuel and clothing, as often happens among the poor in winter, lead to the slower but not less certain disturbance of all the functions, which constitute the less active grades of continued fever. The same view of the action of cold on the elementary vital properties, and through them on the molecular actions in the tissues, will explain the very frequent occurrence of that ephemeral form of fever, usually styled influenza, during the first warm days that follow a week or two of steady and extreme cold weather. But though mental emotions and the imponderable agents may, and sometimes do, exercise a direct influence over the vital properties, sufficient to develop active disease, either general or local, yet they far more frequently serve only to produce that degree of alteration in the properties and actions of the tissues, which constitutes a *predisposition*, and which requires the interposition of some more direct exciting cause to develop the full exhibition of morbid phenomena.

Keeping the forgoing observations in view, let us next examine the symptoms which characterize the first, or forming stage, of the different varieties of fever. All writers agree that this stage of the continued types of fever, including the typhus and typhoid, generally commences insidiously, and progresses so gradually, that a large majority of the patients can fix no definite hour, or even day, when their sickness commenced. Perhaps the earliest and most constant symptom, is a feeling of illness, obtuseness, or indisposition, without the patient being able to say definitely in what the indisposition consists. This soon becomes associated with dull, wandering pains in the head, back and limbs; a sense of weariness; alternate sensations of heat and cold; dryness, and an unnatural taste in the mouth; the mind is dejected; the sleep disturbed and unrefreshing; the appetite lost, or exhibiting some morbid peculiarities; the bowels sometimes feeling heavy and motionless, at other times disturbed, with two or three watery evacuations every day, the latter more especially in typhoid cases; and all these symptoms may continue from three days to three weeks before the patient becomes wholly confined to his bed. Where the causes which lead to the disease exist in a high degree of intensity, however, as is frequently the case on crowded emigrant ships, in jails, camps, &c., the attack is often much more sudden and decisive, and under such circumstances generally assumes the character of a grave typhus. During the last two or three years, I have taken much pains to study the first stage of the continued forms of fever, both in hospital and private practice. And in all those cases which occur sporadically and pass through a protracted forming stage, I have found the pulse early, and pretty uniformly, slightly increased in frequency, but diminished in fullness and force; the capilary circulation retarded, as indicated by a cool and dingy state of the skin, and a slow return of blood after having been removed by pressure; a husky dryness of the surface, dryness of the mouth and fauces, and diminished secretion of urine. The inspirations, too, are less full and forcible, with occasional sighing, and a downcast or stolidly indifferent expression of countenance. The production of animal heat is also diminished in the very incipient stage of the disease, and in some cases it continues so until distinct chills are developed, while in others it gradually gives place to the more decided febrile phenomena without any appreciable chill or shivering.

On what do all these symptoms depend? What is the primary link, the starting point of morbid action? Can they be traced to a primary morbid impression on any one organ or tissue? It is true that most British and American writers refer these incipient phenomena to an impression of a depressing character on the nervous system. But well has Dr. Wood observed that there is something here "more than mere nervous depression." Indeed, almost the only symptoms referrible directly to the nervous tissue, are the local pains, which are more inconstant and variable than any others in the catalogue. That early, though vague, consciousness of indisposition, so far as my examinations go, is invariably accompanied by, and, I think, immediately dependent on, an altered or disturbed state of three universal functions, viz.: nutrition, capillary circulation, and the production of animal heat. And this disturbance consists in depression or retardation, consequent on a diminution of the elementary *susceptibility* of the whole organized matter of the body.

This property and these functions being elementary and common to all the tissues, we readily see how their disturbance would simultaneously involve that universal functional derangement, so graphically described many years ago by Dr. Fordyce.

The susceptibility of the primary cells and fibres of which the tissues are composed, being diminished, that peculiar affinity by which they elect new particles of matter, and reject those that have become effete and useless, is diminished in a corresponding degree. This necessarily involves more or less disturbance of the ultimate capillary and intestitial circulation, and the production of animal heat; and this again involves a change in all the secreting structures, the nerves, the muscles, and every other organized tissue; not excepting the corpuscles of the blood. This depressed condition of the various functions, soon results in the accumulation of offending particles in all the tissues, and an imperfectly depurated state of the blood, which excites the circulating system to an action increased in frequency, but impaired in force; while the diminished exhalation from the pulmonary and cutaneous surfaces lessens greatly the amount of caloric rendered latent, and thereby allows it to accumulate to an uncomfortable excess in the system. And here the second or febrile stage has fairly commenced, exhibiting, instead of those symptoms described in the forming stage, a quicker pulse, a dryer and generally hotter skin, more dryness of the mouth and fauces, a dull, watery expression of the eye, a deeper redness of the face, less muscular strength and steadiness, more decided pains in the head and back, more obtuseness of the intellect and special senses, and a more frequent and decided disposition to local determinations of blood, and the consequent development of local affections in the brain, chest, or abdomen.

The rapidity with which the symptoms are developed, the particular grade of morbid action or alteration of the elementary properties, and the special local determinations in such cases, will depend on the nature and intensity of the cause or causes, the season of the year, and the age of the patient. A very numerous class of cases occur in the large cities, both of this country and Europe, among those who have recently moved from the rural country districts to the more crowded streets of the city. The change in diet and drink, and above all, the inhalation of a less pure and bracing atmosphere, often rendered worse by the occupancy of small and ill-ventilated apartments, undoubtedly induces in such persons, a modification in the elementary properties and functions of the whole system, through their influence on the quality of that universal stimulator of organic action, the arterial blood. In such persons the attack is generally insidious, and the forming stage protracted; but the changes induced by the causes enumerated, may be so rapid as to develop more suddenly the active phenomena of disease. And on the other hand, they may be so slow, that the functions of nutrition, metamorphosis, and calorification, gradually become adjusted to the new impressions, and acclimation takes place without any open morbid action. Another numerous class of cases occur among the poor, with whom an insufficient diet and clothing, co-operates with filthy, ill-ventilated, and damp dwellings, to depress the vital susceptibility, and develop a similar, though perhaps not identical, train of morbid action with that just described. In this class we find almost every conceivable grade of intensity and rapidity of morbid action. When the morbific causes exist and act in moderate intensity, as is usually the case in rural districts, and among the better class of recent residents of cities, the development of febrile phenomena is slow, the several stages of the disease protracted but moderate, and a fatal termination seldom takes place, except through the supervention of some local inflammation or visceral disease. If the attack comes on between the ages of eighteen and thirty years, when the digestive apparatus is most active; and if, in addition, it occur in the latter part of summer and autumn, when the well-known influence of the season has predisposed to gastric and intestinal determinations, it will almost always assume the form of fever, technically styled *typhoid*, with congestion, inflammation, and ultimate ulceration of the glandular structures in the mucous membrane of the small intestine. On the other hand, if the attack occurs in the earlier or later periods of life, when the cerebral and respiratory functions are predominant, or during the cold season of the year, when a low temperature has previously depressed the general susceptibility and diminished the cutaneous excretion, the specific grade of febrile action, when developed, will be lower, and the local determinations more directly to the brain or thoracic viscera. When these two classes of cases are distinct and well marked, they are easily distinguished from each other. The first is generally recognized as the typhoid fever of authors, and corresponds strictly with the common autumnal fever of New England and all the non-malarious sections of our country; while the second, is the sporadic *typhus*, that infests the narrow, filthy streets, and the damp, uncomfortable dwellings of the poor in our cities, and not unfrequently shows itself under the name of *winter fever* in country districts. But I am satisfied, from the most careful clinical observation, that the two varieties merge into each other by such insensible gradations, that every practitioner of large experience, must have seen many cases, which it is impossible for him, from the symptoms during life, to rank positively in one class or the other. So true is this, that the most decided advocates of the separate nature of typhoid and typhus fevers, like Louis, Jenner, Flint, &c., are obliged to leave a considerable number of cases on the *doubtful list*, and even then, occasionally correct a diagnosis that they supposed certain during life, by a post-mortem examination.

When the continued forms of fever arise from the prevalence of some unknown epidemic influence, or the highly poisonous atmosphere generated in crowded ships, camps, jails, hospitals, &c., the primary morbid impression is made on the same elementary properties, and results in the disturbance of the same functions, but the primary lesion is generally more profound, the onset of the disease more sudden, and its whole course attended with more danger. If we compare with the foregoing, the essential phenomena of fevers of the periodical class, we shall find certain analogies and differences, no less interesting than important. In the first place, periodical fevers, unlike those of the countinued types, prevail so uniformly in certain districts of the country and at certain seasons of the year, that they almost irresistibly fix in the mind of the observer the idea that their efficient cause is some specific agent generated within such districts and incapable of generation elsewhere. But whatever may be the nature or origin of the efficient cause, its *modus operandi* is the more immediate object of our present study. To pursue this study with the least liability to error, let us again take our station by the bed-side, and observe the initial phenomena, that usher in the more active stage of these fevers. Though the attacks of intermittents and remittents are much more sudden and decisive than those of the continued forms, yet, I never recollect to have met with a patient who did not complain of a forming stage varying from one to three or four days. "In general," says Dr. Drake, "a remittent is preceded by a forming stage of one, two, or three days, in which there is an increasing languor of the muscular system, inefficiency of body and mind, defective perspiration, rigors, sometimes alternating with flashes of heat, a torpid state of the bowels, increased or diminished secretion of bile, a bilious hue of the eyes, loss of appetite, a foul and generally white tongue, having sometimes a tint of yellow; and, in most instances, a dull pain in the head and back."—Again the same eminent author makes the first stage of an intermittent consist in a "reduction of vital energy, obtuseness of sensibility, suspended or perverted secretion, and diminished calorification." And these phenomena he attributes to the influence of a specific agent, floating in the blood, and possessing "a sedative and irritating quality, somewhat like the narcotico-irritating gases." But here, too, Dr. Drake, like Dr. Wood, follows the lead of Dr. Southwood Smith, and makes the function of *innervation* the first affected. What this term innervation] includes, as used by authors, is not very clearly defined. If it refers to that peculiar influence which emanates from the brain, medulla oblongata, spinal cord, or ganglionic centres, to the various muscular structures, including the voluntary and involuntary or organic, in response to impressions received from the different surfaces of the body; then, certainly, it is less early and profoundly affected than several other functions. Indeed, according to my own observations, the evidences of disturbed nutrition, calorification, and secretion, invariably precede the symptoms of nervous disturbance. These evidences consist in a variableness or loss of appetite, an unnatural taste in the mouth, with some coating of the tongue, frequent sallowness and dryness of the skin, scanty and high colored urine, variableness of temperature, and a universal feeling of langour or indisposition to exertion. To trace minutely and clearly the thread or chain of morbid action, I should assume that some agent, which constitutes the specific or efficient cause of periodical fever, had entered the blood, and by its presence throughout the whole organism. it so modifies the vital affinities, that the tonicity or texture of the solids becomes universally impaired, while the susceptibility remains normal or it is morbidly increased.

This interference with the tonicity and vital affinity of the solids at once retards or suspends those processes by which assimilation and nutrition are effected and animal heat developed; and also by which the secreting cells of the excretory organs are enabled to select from the blood, the effete particles which constitute their respective secretions. By this change, the formation of new corpuscles in the blood ceases; the disturbance of nutrition, metamorphosis, and calorification throughout the brain, the nerves, the muscles and everywhere, causes the general feeling of the lassitude and indisposition; while the retarded or perverted action of the secreting structures soon accumulates an amount of retained effete matter or perverted secretions, or both, which not only explains the loss of appetite, the coated tongue, the epegastric uneasiness, the scantiness of the urinary and cutaneous secretions &c., but which also directly co-operates with the irritant quality of the original specific cause, in exciting the elementary susceptibility to a much higher degree. It is while these primary perturbing changes are taking place in the tissues, that the nervous and muscular systems pass through that peculiar period of agitation which constitutes the chill, and which always terminates where the vital susceptibility becomes so much increased as to predominate in its effects over those arisng directly from the impaired tonicity. Hence the more irritating the qualities of the specific cause, and the more rapid the consequent accumulation of perverted secretions, the shorter and less marked the chill, and the more persistent the febrile excitement, constituting the *remittent* variety of the disease. On the other hand, when the sedative quality of the cause predominates and it acts with moderate intensity, the tonic y of the tissues and the primary organic functions are impaired rather than perverted; the capillary circulation and calorification remain longer depressed; and consequently the chill is more perfect and protracted, while the succeeding hot stage is less violent and of shorter duration, constituting the ordinary intermittent. But there are other cases again in which the sedative quality of the cause so strongly predominates, and it acts with such intensity as to profoundly impair both tonicity and susceptibility, leading not simply to a chill, but to an absolute suspension of capillary circulation, secretion and calorification, constituting the true *malignant* or pernicious form of periodical fever, in which the reaction or hot stage supervenes slowly and imperfectly, or not at all. And there are still other cases, in which the essential cause acts so freely that active febrile phenomena are never developed, but its pernicious effects, are seen in the altered hue of the skin, the diminished energy and the morbid susceptibility of the patient. In all cases of simple periodical fever, when the hot stage commences, the organic susceptibility becomes so rapidly exalted that the heart and arteries take on a high degree of activity, the capillary circulation and production of animal heat become correspondingly active, accompanied by great restlessness, pains in the back and head a morbid acuteness of sensibility and of the special senses, and frequently, great increase of some secretions, with diminution of others. Here we see the marked contrast between the strictly periodical and continued forms of fever. In the first, tonicity is impaired while susceptibility becomes so much exalted as to induce the highest grade of irritative action throughout the entire organization. In the latter *susceptibility* is directly impaired, inducing throughout the whole course of the disease, obtuseness of sensibility and sluggishness of organic action; while the tonicity often remains so little impaired that patients will not unfrequently rise from the bed and walk, within a few hours of a fatal termination. Here, too, we find an explanation of their widely different terminations and sequalæ. The high irritative organic excitement of the one cannot long continue without leading either to universally increased secretion, constituting the sweating stage, to be followed by a more or less perfect intermission, or to local determinations, congestions, inflammations, and in the sequel to impoverished blood, impaired textures, and œdematous or dropsical effusions. While the impaired susceptibility, the enfeebled capillary circulation, the obtuseness of sensibility, and the sluggishness of the organic movements of the other, lead to a far more protracted and uniform continuance of morbid action, with less active and varied local determinations, and a greater ultimate tendency to softening and ulceration. The direct impairment of tonicity and vital affinity, coupled with the irritative excitement arising from exalted susceptibility, which constitutes the periodical form of fever, produces a more complete suspension of that part of the assimilative process by which the blood corpuscles are formed, and hence more directly and certainly leads to a marked impoverishment of the blood and impairment of the solid textures, than almost any other disease with which we are acquainted.

The three following cases and analysis are selected from my note book, for the purpose of illustrating the changes which the blood undergoes, while the elementary properties and functions of the solids are disturbed in such a manner as to constitute idiopathic fever. I have chosen to give, in this place, a very few cases in detail, rather than any general statement of the average results of many analysis, that the reader may see in their proper connection, the particular symptoms and stage of each case, with the accompanying changes in the circulating fluid. We have already, scattered through the literature of our profession, many general statements of the changes of the blood in different forms of disease, but very few of them contain any account of the particular symptoms, or stage of the disease, when the blood was taken. Hence much of their value, in a pathological view, is lost.

### Case 1.

Peter Johnson, a Swede, aged thirty-five years, was admitted into the Illinois General Hospital, November 27th, 1852.

(*Continued next month.*)

